# Nano Drug Delivery Platforms for Dental Application: Infection Control and TMJ Management—A Review

**DOI:** 10.3390/polym13234175

**Published:** 2021-11-29

**Authors:** Abhishek Lal, Mohammad Khursheed Alam, Naseer Ahmed, Afsheen Maqsood, Ruba K. Al-Qaisi, Deepti Shrivastava, Zainab Ali Alkhalaf, Amal Mohamed Alanazi, Hasna Rasheed Alshubrmi, Mohammed G. Sghaireen, Kumar Chandan Srivastava

**Affiliations:** 1 Department of Prosthodontic, Altamash Institute of Dental Medicine, Karachi 75500, Pakistan; abhishekdarshan@yahoo.com; 2 Orthodontics, Department of Preventive Dentistry, College of Dentistry, Jouf University, Sakaka 72345, Saudi Arabia; 3 Department of Dental Research Cell, Saveetha Dental College and Hospitals, Saveetha Institute of Medical and Technical Sciences, Chennai 600077, India; 4 Prosthodontics Unit, School of Dental Sciences, Health Campus, Universiti Sains Malaysia, Kota Bharu 16150, Malaysia; 5 Department of Oral Pathology, Bahria University Dental College, Karachi 75530, Pakistan; afsheenmaqsood@gmail.com; 6 Prosthodontic Dentistry Department, Jordanian Royal Medical Services, Amman 11180, Jordan; rkqaisi@gmail.com; 7 Periodontics, Department of Preventive Dentistry, College of Dentistry, Jouf University, Sakaka 72345, Saudi Arabia; 8 Prosthodontic Dentistry Department, College of Dentistry, Jouf University, Sakaka 72345, Saudi Arabia; dr.zainab.alkhalaf@jodent.org (Z.A.A.); amal-6677@hotmail.com (A.M.A.); dr.hasna.alshubrmi@jodent.org (H.R.A.); dr.mohammed.sghaireen@jodent.org (M.G.S.); 9 Oral Medicine & Radiology, Department of Oral & Maxillofacial Surgery & Diagnostic Sciences, College of Dentistry, Jouf University, Sakaka 72345, Saudi Arabia; drkcs.omr@gmail.com; 10 Department of Oral Medicine & Radiology, Saveetha Dental College and Hospitals, Saveetha Institute of Medical and Technical Sciences University, Chennai 600077, India

**Keywords:** nanoparticles, drug delivery systems, oral infections, temporomandibular joint, nanomedicine, COVID-19

## Abstract

The oral cavity is an intricate environment subjected to various chemical, physical, and thermal injuries. The effectiveness of the local and systemically administered drugs is limited mainly due to their toxicities and poor oral bioavailability that leads to the limited effectiveness of the drugs in the target tissues. To address these issues, nanoparticle drug delivery systems based on metals, liposomes, polymeric particles, and core shells have been developed in recent years. Nano drug delivery systems have applications in the treatment of patients suffering from temporomandibular joint disorders such as preventing degeneration of cartilage in patients suffering from rheumatoid arthritis and osteoarthritis and alleviating the pain along with it. The antibacterial dental applications of nano-drug delivery systems such as silver and copper-based nanoparticles include these agents used to arrest dental caries, multiple steps in root canal treatment, and patients suffering from periodontitis. Nanoparticles have been used in adjunct with antifungals to treat oral fungal infections such as candida albicans in denture wearers. Acyclovir being the most commonly used antiviral has been used in combination with nanoparticles against an array of viral infections such as the herpes simplex virus. Nanoparticles based combination agents offer more favorable drug release in a controlled manner along with efficient delivery at the site of action. This review presents an updated overview of the recently developed nanoparticles delivery systems for the management of temporomandibular joint disorders along with the treatment of different oral infections.

## 1. Introduction

**Figure 1:**
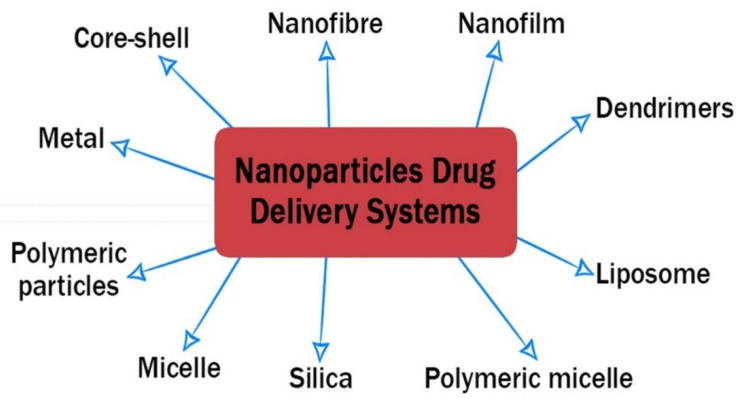
Different forms of nanoparticles used currently in dentistry.

The oral cavity is an intricate environment that is subjected to various chemical, physical, and thermal injuries. The oral cavity is also a site of infections and pathological changes to the temporomandibular joint (TMJ). Many bacterial, viral, and fungal infections are found affecting the oral cavity such as dental caries, periodontitis, endodontic diseases, candidiasis, herpes simplex virus, and varicella zoster infection [1]. Temporomandibular joint disorders are prevalent worldwide which can cause various morbidities for the patients suffering from it. TMJ disorders are also associated with other systemic disorders such as rheumatoid arthritis, and osteoarthritis [2]. The traditional therapy of using locally and systemically administered medications may not be effective enough due to the lack of absorption of the drugs in sufficient amounts along with disturbances caused by constant salivary flow [3]. Furthermore, administration of drugs in this manner also leads to side effects that are at times disturbing for the patient. To avoid such unnecessary circumstances, in the past few decades’ various new techniques of drug administration have been worked upon to address its dental applications such as infection control and patients with TMJ problems [4]. One of the popular techniques is using nanoparticles incorporated agents that can be used which ensures safe and effective delivery of the drugs to the required affected areas [5]. Nanoparticles drug delivery offers various more advantages such as avoiding drug-drug interaction, side effects, increasing bioavailability, and selectivity. At the present moment, nanoparticles are being used to treat various dental diseases but the frequency with which it is being used varies considerably. Nanoparticles are available in various different kinds of structures such as nano-capsules, nano-spheres, mesoporous nanoparticles, and core-shell nanoparticles [6,7,8,9] as presented in Figure 1.

**Figure 2:**
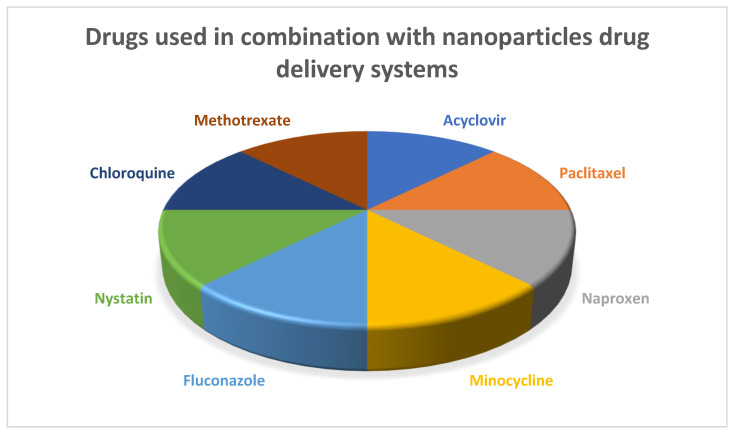
Commonly used drugs in combination with nanoparticles drug delivery systems.

Different types of nanoparticles combinations are used in the drug delivery systems such as silver nanoparticles, copper nanoparticles, and chitosan-based nanoparticles along with lipid-carrier-based nanoparticles [10]. Each of these nanoparticles has different applications in dentistry such as being antibacterial, antiviral, anti-inflammatory, and antifungal agents, as presented in Figure 2. Hydrogels, nanofibers, thin films, and other nanocomposites have also been used in dentistry [11]. However, nanoparticles have been incorporated into hydrogels that improve their mechanical and optical properties [12]. Nanoparticles and nanofibers have also been studied in literature with bovine serum albumin (BSA) being used as an active pharmaceutical agent for comparison [13]. This study concluded that a larger quantity of BSA was released from nanoparticles than that from nanofibers, which indicates a greater degree of drug adsorption on the surface of the nanoparticles during the process of encapsulation [13]. Nanoparticles and thin films have been used in combination in one study which report the combination of silver nanoparticles into polymethylmethacrylate thin films where higher microbicidal activity was found [14].

## 2. Temporomandibular Joint Disorders

**Figure 3:**
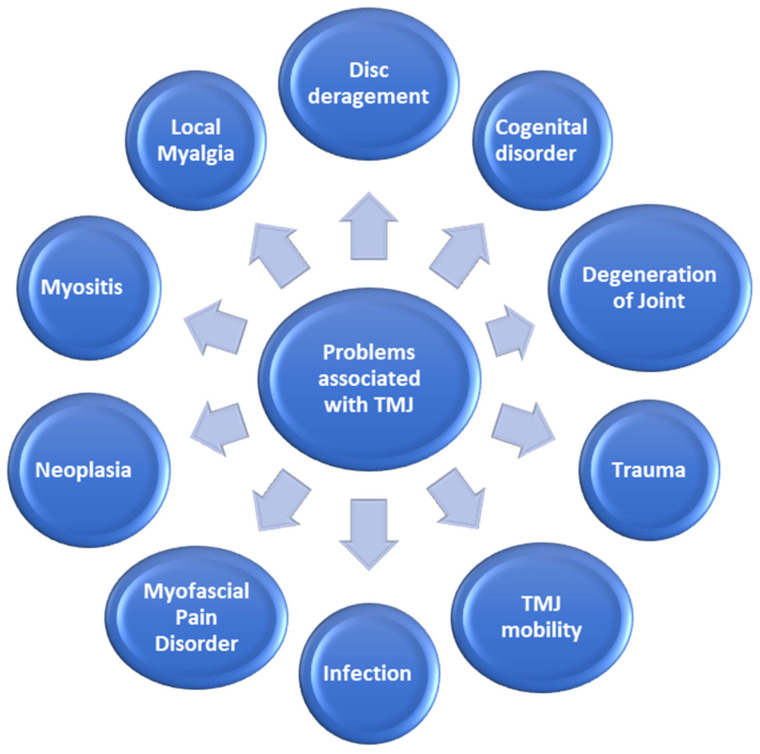
Different problems that are associated with TMJ.

The temporomandibular joint is one of the most vital joints of the human body as it is involved in performing important functions such as guiding the mandibular movements and regulating the stresses that are produced throughout the day such as mastication, speech, and deglutition [15]. About the anatomy of the TMJ, it is a diarthrosis or more precisely termed as ginglymoarthrodial joint which is composed of a synovial cavity, articular cartilage, and capsule that covers the entire joint [16]. TMJ is a joint that is commonly associated with many pathologies that can either be localized to the joint or associated with other systemic pathologies such as rheumatoid arthritis and osteoarthritis [17], as presented in Figure 3.

### 2.1. Preventing Cartilage Degeneration of TMJ

Degeneration of the TMJ cartilage is among the common features in patients suffering from osteoarthritis, rheumatoid arthritis, and psoriatic arthritis, which then further leads to jaw pain and limited movements that are functionally debilitating [18,19,20]. Currently, patients suffering from these pathologies are treated with steroid injections, pulsed electrical stimulation, pharmacological drugs, hyaluronic acid injections, and topical ointments along with stem cell therapy, which provides relief to various degrees to patients, respectively [21,22].

Nanoparticles are present in various different sizes in nanometers which readily enter the target cells by endocytosis mechanism. Biosynthetic molecules such as poly lactic co-glycolic acid (PLGA) and poly lactic acid (PLA) or chitosan and gelatin (natural polymers) are used to make nanoparticles [23]. Chondrocytes are the main target cells for the regeneration of the TMJ cartilage. Hyaluronic acid (HA) which is part of the connective tissue, is incorporated in the nanoparticles, which target CD44 receptors that are expressed on the chondrocytes which facilitate TMJ cartilage regeneration, being the vital cells in regeneration [24]. Using HA/chitosan nanoparticles, a non-viral gene was delivered which was successful in transforming an exogenous gene into chondrocytes (primary) that led to the protection of the cartilage from degeneration [25].

Reactive oxygen species (ROS) are also responsible for inducing inflammatory reactions that lead to cartilage degeneration. So, therefore, targeting these ROS via nanoparticles can be beneficial in protecting the TMJ cartilage. Nanoparticle’s antioxidants have been purposed as options for such cases [26].

### 2.2. Anti-Inflammatory Effects

Inflammation of the temporomandibular joint is a frequent pathology that is suffered by many patients, and it affects adjacent tissues of the joint as well. Most commonly, such patients are treated with non-steroidal anti-inflammatory drugs (NSAIDs). When taking the NSAIDs in a conventional way such as oral administration, to reach the desired level of efficacy required, it can take many days or weeks since they are administered systemically and required repeated doses [27].

**Figure 4:**
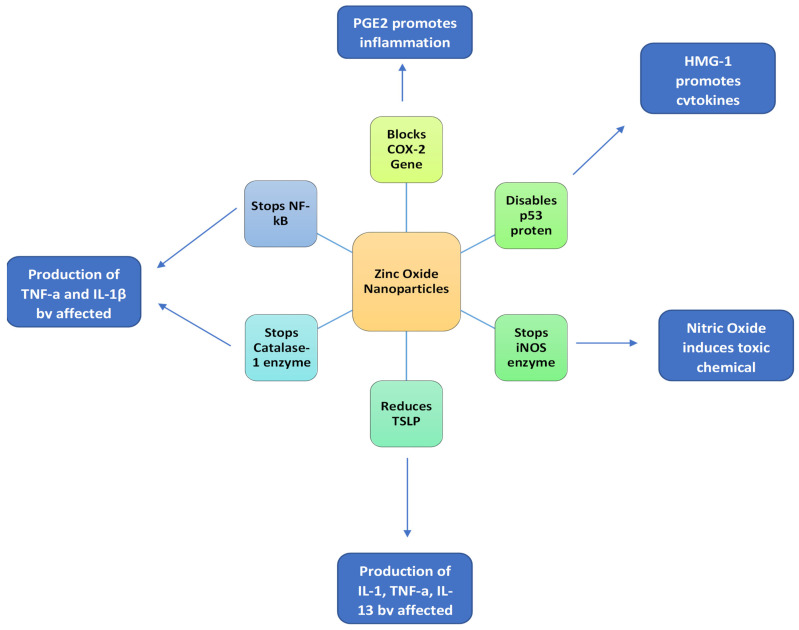
Anti-inflammatory mechanism of ZnO nanoparticles. Cox-2 = cyclooxygenase enzyme 2; IL = interleukin; TNF-α = tumor necrosis factor alpha; TSLP = thymic stromal lymphopoietin; NF-kB = nuclear factor kB; iNOS = inducible nitric oxide synthase.

To enhance the beneficial properties of the NSAIDs, naproxen is incorporated in nanostructured lipid carriers (NLC) for intra-articular injection for the reduction of inflammation in TMJ disorders [28]. Naproxen and a nanostructured lipid carrier were used in combination where naproxen was the active molecule in the carrier system with successful results obtained from rats [28]. Naproxen mainly targets cyclooxygenase-1 and 2 (COX) which causes the production of PGE2 which then further leads to inflammatory reactions. Now, intra-articular injection although beneficial by targeting the joint directly as some disadvantages associated with it such as joint pain due to volume of injection and particular lack of patient compliance [29]. To counteract such limitations, the nanoparticles drug delivery system has been proven to be advantageous. These drug delivery systems enhance delivery, reduce systemic toxicities, and stability of the drugs [30]. Figure 4 presents the anti-inflammatory mechanism of zinc oxide (ZnO) nanoparticles.

Methotrexate (MTX) based nanoparticles have been developed in combination with NLC to treat patients with rheumatoid arthritis presenting frequently with TMJ disorders. In such cases, the swelling had significantly reduced during a period of 28 days when administered in rats [31]. Paclitaxel, an antineoplastic drug used in cancer chemotherapy, interferes with several processes involved with inflammatory arthritis such as angiogenesis and collagenase expression but its use is limited mainly due to systemic toxicity. To overcome such limitations, Paclitaxel, in combination with phospholipid mimetic nanocarrier system is used as an intra-articular injection in rat joints results in significant improvement in gait scores and inflammation in synovial joints, with a confirmed reduction in TNFα levels [32]. Phospholipid nanocarrier system was prepared using dipalmitoyl-sn-glycero3-phosphotidylcholine (DPPC) prepared using a thin film hydration method with a size of 311 ± 57 nm and 92 ± 0.6% paclitaxel encapsulated with short initial burst phase and sustained release profile with a cumulative release of 18 ± 0.36% [32]. Thymol, a natural-synthetic compound, has several benefits associated with it such as being antibacterial, antiseptic, and antioxidant but they decompose easily. When combined with NLC, thymol was found to be stable, with reduced systemic toxicities with improved anti-inflammatory effects with psoriatic mouse model along with improved healing [33].

Nanolipid carriers-naproxen (NLC NPX) efficacy in reduction of inflammation was measured by leukocyte infiltration in TMJ of rats. In the initial first 10 days of injection of NLC NPX, the leukocyte infiltration was reduced but after the 10th day, the effect had abrogated [34].

### 2.3. Anti-Arthritic Efficacy

Arthritis affects many joints in conditions such as rheumatoid arthritis, especially the temporomandibular joint. Methotrexate is primarily used as an antiarthritic drug to control ongoing arthritic effects. Recently, in many in vivo studies, methotrexate has been incorporated in nanolipid carriers to target specific joints only. In comparison with the conventional way of taking methotrexate, when MTX is combined with NLC, superior anti-arthritic effects were noted which reduced inflammation significantly [35].

Anti-arthritic effects of hesperidin (HP) incorporated in gum acacia (GA) with silver nanoparticles (AgNP) has been investigated. HP loaded with GA-AgNP should reduction in swelling and degenerative changes with the primary mechanism of action by interfering with Toll-like receptors (TLR-2 and TLR-4) mechanism which aggravates arthritis [36].

Chitosan-based nanoparticles (CS-NP) have been used as subcutaneous injection as an anti-arthritic treatment modality in rheumatoid arthritis patients. Chitosan-based nanoparticles were prepared using the ionotropic gelation method where 1 g chitosan was fully dissolved in 50 milliliters glacial acetic acid 1%, mixed at 40 degrees to achieve a clear solution with nanoparticles having a size of 102.4 ± 3.2nm. Rats were induced with arthritis that showed a significant decrease in malondialdehyde (MDA) and nitric oxide (NO) levels which resulted in the return of normalcy of antioxidants [37]. Furthermore, inflammatory mediators such as TNFα, IL-1, IL-6, and IL-1B concentration in serum were reduced by administration of CS-NPs [37].

## 3. Oral Infections Management

### 3.1. Antibacterial Effects

#### 3.1.1. Dental Caries

Biofilms in the oral cavity can cause many diseases such as dental caries and periodontal disease [38]. Dental caries is a demineralization disease of the hard tissues of the teeth such as enamel and dentin, which if left untreated, eventually affects the pulp [39]. On the other hand, periodontal disease is associated with effects on the periodontal structures of a tooth such as gingiva, cementum, alveolar bone, and periodontal ligaments. Traditionally, both of the disease entities have been targeted with mechanical plaque control methods, which causes biofilm formation [40,41]. Many different kinds of methods have been developed that primarily focus on preventing dental caries along with arresting dental caries. Regular use of a toothbrush with toothpaste containing fluoride twice per day along with the use of dental floss has been the recommended strategy to prevent and arrest dental caries [42].

Nanoparticles can incorporate within many antimicrobial and antiplaque agents for better treatment, as presented in Table 1.

**Figure 5:**
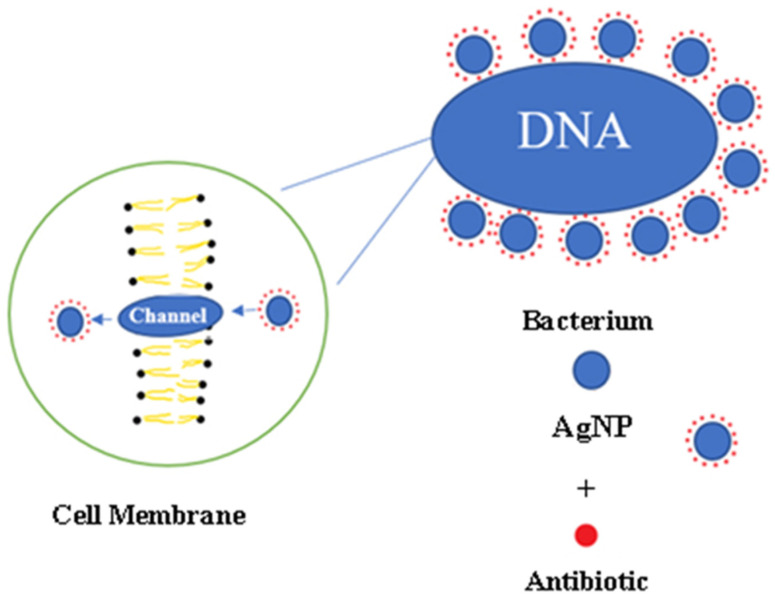
Antibacterial agents’ combination with nanoparticles.

Silver and copper have been the most studied metals with their antimicrobial properties used in combination with nanoparticles [43,44]. As presented in Figure 5 Silver and copper-based nanoparticles offer excellent antibacterial properties primarily due to their high surface area to volume ratio that enables greater accumulation of atoms on its surface, thereby providing maximum contact with the environment [45]. Due to their smaller size, these small particles can easily and sufficiently cross the cell membranes that eventually increasing reactivity and antibacterial effects [46].

Silver nanoparticles have been used in a variety of situations such as coating of implants and dressing of wounds [47]. One study in the literature concluded that the antimicrobial action of the silver nanoparticles is mainly associated with the number of silver ions that are released along with its interaction with the cell membranes of the bacteria [48]. Silver nanoparticles have been incorporated in various different dental materials to counteract the action of the bacteria in preventing and arresting dental caries. A study by Jurgen et al. incorporated silver nanoparticles in dental composite to evaluate its antibacterial [49]. These substances also have been incorporated in different materials such as hydrogels and polymethylmethacrylate (PMMA) [50]. The oral cavity is abundant with bacteria such as *Streptococcus intermedius, Porphyromonas gingivalis, Fusobacterium nucleatum, Prevotella intermedia,* and *Aggregatibacter actinomycetememcomitans*, all have been found to be susceptible to copper oxide and silver nanoparticles [51]. Different in vitro studies have successfully concluded that the addition of silver nanoparticles in the dental composite did not significantly compromise the mechanical as well as esthetic properties of the dental composite [52,53]. These findings suggest the possible use of silver nanoparticles incorporated dental composite for their antibacterial action. Moreover, one study previously has suggested prolonged antibacterial activity of the silver nanoparticles mainly due to prolonged silver ions release [54].

#### 3.1.2. Endodontic Diseases

**Figure 6:**
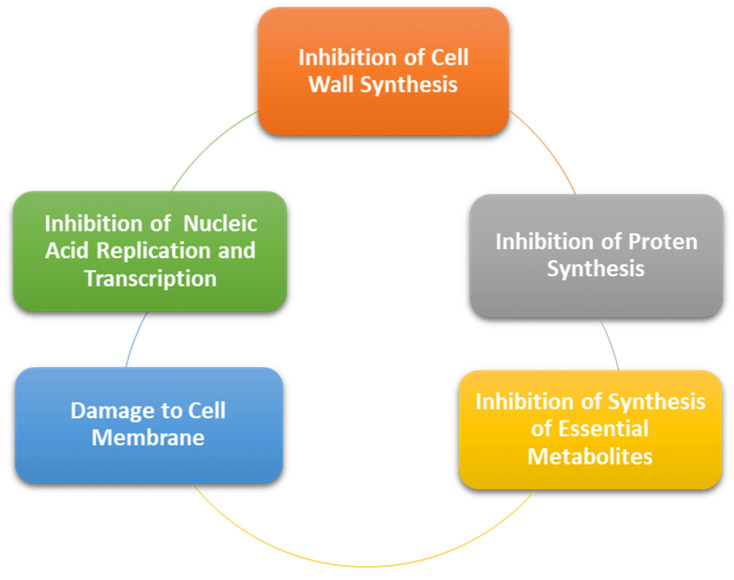
Mode of action of antibacterial agents.

Considering endodontic diseases, it encompasses various pathologies from vital pulp to a necrotic pulp with cellulitis and abscesses. To treat such diseases, root canal therapy is most commonly performed in association with antibacterial agents such as intracanal medicaments and systemic antibiotics [55]. Most of the antibiotics have a similar mode of action, as presented in Figure 6. In regards to the complex anatomy of the root canal systems, such traditional treatment strategies are not useful all the time. For such cases, nanotechnology can be proven to be beneficial. Nanoparticles can be incorporated within sealers, intracanal medicaments, obturation materials, and irrigation solutions [56]. One study has found that silver nanoparticles when used in combination with calcium hydroxide have shown to have increased antibacterial activity as compared to calcium hydroxide used alone or with chlorhexidine [57]. Obturation is one of the vital parts of root canal therapy with gutta percha (GP) being the most commonly used material obturation. A study by Lee et al. concluded that the use of nanoparticles with bioglass as Nano-diamond GP composite embedded with amoxicillin showed greater mechanical properties such as elastic modulus and strength [58]. Sealers have been used in root canal therapy with GP in order to fill the voids and achieve a tight three-dimensional seal. A study by Kishen et al. incorporated zinc oxide and chitosan in sealers and found that the nanoparticles were successful in their antibacterial action by preventing the penetration of bacteria into the root canals [59]. Moreover, another study concluded that endodontic sealers incorporated with chitosan nanoparticles were efficient in their antibacterial activity for a long period of time [60]. Antimicrobial photodynamic therapy (aPDT) has been used in combination with nanoparticles with in-vitro studies testing its efficacy against *Enterococcus faecalis* in extracted human teeth. Successful reduction in colony-forming units (CFU) was noted when aPDT nanoparticles were used as compared to controls where higher CFU were noted [61].

Chitosan nanoparticles (CS-NP) have been incorporated within the traditionally used sealers, compare its effect with conventional sealers. By using CS-NP, it was found that biofilm formation was inhibited within the dentin-sealer interface as compared to using conventional sealer formulation, after being evaluated for 30 days [60].

#### 3.1.3. Periodontal Diseases

Patients suffering from various periodontal conditions require not only mechanical plaque removal, but administration of antibiotics as well. Since most of the antibiotics are given systemically, they lead to unwanted side effects, limited permeability of drugs into the target cells, and at times bacterial resistance as well. So, to avoid such circumstances, local delivery of drugs within the periodontal pockets can overcome such disadvantageous scenarios. Studies do report transportation of the nanoparticles within the junctional epithelium using an intra-pocket delivery system [62].

Chitosan nanoparticles either with PLGA or polyvinyl acetate have been used to treat periodontitis-causing bacteria. When placed inside the periodontal pockets, its antibacterial properties were noted with a reduction in the bacterial count even in the constant presence of salivary stimulation [63].

Minocycline incorporated PEG-PLA nanoparticles have been studied in the periodontal pockets with noted sustained release in vitro over the course of 14 days with the drug remaining effective against the bacteria for over 12 days within significant reduction in the periodontal symptoms, as compared to the control [64].

Subgingival plaque and calculus are known to be one of the primary factors in causing gingivitis and then untreated gingivitis may lead to periodontitis. Such plaque and calculus are not cleaned by the use of toothbrush and requires treatment by the dental surgeons. A study by Hayakumo et al. concluded that use of ozone nanobubble that is produced by nanobubble technology can the used as subgingival irrigation for its antibacterial properties [65].

Host modulation therapy has been an emerging field of interest to the researchers that reduces and even stabilizes or might even regenerate inflammatory tissues by modifying the host response [66]. Host modulation therapy has been extensively studied in periodontics. One study by Cafferata et al. reported the efficiency of use of nanocarriers such as PLGA, chitosan, and silica-derived nanoparticles for treating periodontal conditions [67]. This study found the immunomodulatory effects of host modulating agents delivered by nano drug delivery systems. Levels of proinflammatory cytokines such as interleukin-1, TH-22, TH-17, and TH-1.

### 3.2. Antifungal Effects

Candida albicans, is the primary fungal infection that targets the oral cavity. Conventionally, many antifungal medications such as nystatin and azoles, both systemically and locally, have been employed to tackle the infection but with unwanted side effects as well. Nanoparticles have been incorporated with copper, silver, and palladium, to inhibit the growth of many microorganisms such as *candida albicans* [68]. Used as an antifungal agent, it is capable of doing cell wall damage, increase in oxidative stress, and interaction with DNA [69]. Silver nanoparticles have been used as an antifungal agent.

Denture wearers frequently complain of suffering from *candida albicans* induced infections due to prolonged wear. So, nanoparticles have been successfully incorporated into PMMA to combat such infections. Silver nanoparticles have been shown to decrease microbial colonization over the prosthesis [70]. An in vitro study of Artemisia annua produced AgNP have shown antifungal activity against three Candida species such as *Candida glabrata*, *Candida tropicalis*, and *Candida albicans* [71].

Zirconium oxide nanoparticles (ZrO2 NP) incorporation into PMMA has shown to increase the density of the prosthesis along with a decrease in porosity which is responsible to enhance tensile strength, fracture strength, and flexural strength [72]. Due to the incorporation of ZrO2 NP into PMMA which improved the mechanical properties of the prosthesis, this resulted in a decrease in adhesion and proliferation of *Candida albicans* [73].

Nanoparticles coated with fluconazole have been used using ethylcellulose and Flu- eudragit RS 100 polymers. The results concluded that nanoparticles coated with fluconazole exhibited prolonged release of fluconazole over 90% for a period of 8 h as compared to conventionally used systemic fluconazole with 90% released during the first hour, therefore improved antifungal activity [74].

Nystatin, a very effective antifungal agent used to treat many fungal infections has a very short duration of action in target tissues which limits its use and often requires multiples doses. To overcome this drawback, Nystatin has been incorporated with nanoparticles. Unloaded nystatin was formulated and fully characterized and incorporated into oral gel and toothpaste, with sizes ranging from 300 to 800 nm after loading [75]. It was found that nystatin-coated nanoparticles exhibited prolonged release, high encapsulation efficiency, and high adhesion capacity to the oral cavity as compared to conventionally used nystatin alone [75].

### 3.3. Anti-Viral Effects

The oral cavity is populated by various types of microorganisms for e.g., Streptococcus, Eubacteria, and Fusobacterium, with viruses being one of them such as herpes simplex virus 1 and 2 (HSV-1 and HSV-2). These viral infections are a major global public health challenge, with resistance to major drugs against these viruses another hurdle. So, to overcome this challenge, a nanotechnology drug delivery system has been worked upon.

#### 3.3.1. Acyclovir Based Nanoparticles

Acyclovir is one of the most commonly used antiviral drugs primarily against herpes simplex infections (HSV-1 and HSV-2). When taken orally, one of the limitations of this drug is its poor bioavailability [76]. Nanoparticles have been combined with various drugs to limit their toxicities. For acyclovir, lipid nanoemulsion particles have been used which is stabilized by emulsifier and/or surfactant which coats the nanoparticles. One study report increased oral bioavailability of nanoemulsion systems of acyclovir in rats when compared with commercially used oral acyclovir [77,78]. In this study, it was also reported that the blood circulation time of the nanoemulsion system of acyclovir was prolonged to 3.5-fold increase as compared to the conventional drug. In vitro drug release study of nanoparticles acyclovir loaded with Eudragi RLPO concluded sustained release of the drug for over a period of 24 h [79].

Acyclovir IV nanoparticles injections have also been formulated to increase further the bioavailability and efficacy of the drug. Administration of the conventional IV acyclovir commonly causes thrombophlebitis at the injection site [80]. Furthermore, higher doses can also cause kidney damage due to the deposition of drug crystals in the kidney [81]. So, proper adjustment of the doses is required.

Using polylactic-co-glycolic acid (PLGA), nanoparticles novel IV acyclovir has been formulated to overcome this limitation of poor bioavailability. When acyclovir was combined with PLGA, a nanoparticle size of 200–300nm was achieved [82,83]. When this combination of acyclovir with PLGA was injected, it was found that the biodistribution had significantly improved with the highest levels of it in the liver as compared to other organs such as lungs and kidneys [83]. So, it was concluded that successful use of acyclovir PLGA nanoparticles resulted in targeted therapy and increased bioavailability as compared to conventional drug therapy.

#### 3.3.2. Chitosan-Based Nanoparticles against Viral Infections

Chitosan has been primarily used in medical sciences mainly due to its non-toxicities, biocompatibility, biodegradability, and passing through tight capillary junctions which helps in better penetration into target tissues [84]. Although these nanoparticles drug delivery systems offer many advantages, but they come with higher prices as compared to conventional drugs [85].

HSV-1 is known to affect the oral cavity due to its high transmissibility rate, occurring most commonly during childhood has herpetic gingivostomatitis with recurrent labial lesions. To treat patients infected with HSV-1, many in-vitro studies have been carried on chitosan-loaded nanoparticles. A study by Donalisio et al. found that chitosan-based nanoparticles loaded with acyclovir presented with increased potency against the HSV-1 and HSV-2 viruses as compared to using acyclovir alone with no reported cytotoxicity against the host cells [86].

Interferons are known for the capability to suppress viral replication by inhibiting its translation. A study reports that when chitosan nanoparticles are administered in mice, their splenocytes secreted increased amounts of interferon-gamma [87,88].

#### 3.3.3. Chloroquine Based Nanoparticles against Viral Infections

Chloroquine phosphate (CHQ) has been successfully used to treat many infections such as malaria, and some inflammatory disorders too, such as rheumatoid arthritis and lupus erythematosus. CHQ in combination with nanoparticles drug delivery systems has been used to improve the efficacy of the drug at the targeted tissue site [89,90].

Chloroquine diphosphate in combination with polylactic acid (PLA) nanoparticles (CQ-NP) has been tested to improve its efficacy against HSV-1. It was concluded that the use of CQ-NP significantly improved the antiviral properties of chloroquine diphosphate against HSV-1 [91].

#### 3.3.4. Nanoparticles Drug Delivery System against SARS-CoV-2

In a recent study by Chakravarty et al., the use of lipid-based, metal-based and polymeric substances as nanoparticles for delivery of antiviral drugs in coronavirus disease-19 (COVID-19) patients has been studied [92]. The size of the nanoparticles and the coronavirus is very much similar so it can be successfully targeted by the nanoparticles antiviral drugs thereby providing effective treatment outcomes [93].

Nanoemulsion, due to its favorable properties can be used to carry antiviral drugs [94]. Few studies have studied the mode of action of nanoemulsion on some viruses and they concluded that nanoemulsion can interfere with the viral envelope formation and mask virus (coronavirus) building, which may prevent absorption of the coronavirus and invasion into cells [95,96].

Dendrimers are drug delivery systems that are used to administer insoluble drugs [97,98]. Dendrimers have been loaded with methotrexate with the results of an increase in its concentrations [99]. With studies reporting the antiviral properties of dendrimers, they have become the main candidates against viral infections such as HSV-1 and COVID-19 [100,101]. One study investigated the effects of dendrimers nanoparticles against dangerous pathogens such as influenza and Ebola [102].

## 4. Future Prospective

Nanoparticles drug delivery systems have been studied and researched in the past few decades as it offers many significant advantages over the conventional methods of administering drugs such as increased bioavailability and efficiency of drugs at the site of action and decreased systemic adverse effects of the drugs. At the present moment, nanoparticles drug delivery system has limited application in dentistry that needs further studies to enhance its scope for use in dentistry such as to diagnose and treat oral cancers, use of nanoparticles in mouthwashes and toothpastes as an adjunct in non-surgical periodontal treatments, local anesthesia, and treating dental hypersensitivity.

## 5. Conclusions

At the present moment, the use of nanotechnology especially in dentistry is continuing to expand with its various applications such as in treating temporomandibular joint disorders and oral infections. Nanoparticles offer greater oral bioavailability, few side effects, and increase patient’s compliance when taking drugs in this form. Synthesis of such nanoparticles-based drug delivery systems is playing a pivotal role, especially considering its application in dentistry. Nanoparticles have been used in combination with antibacterials, antivirals, and antifungals to optimize the treatment of dental infections along with temporomandibular joint disorders. In the future, more clinical trials should be conducted to further explore the beneficial effects of nanoparticles drug delivery systems.

## Figures and Tables

**Table 1:** Antimicrobial activity of different types of nanoparticles.

Types of Nanoparticles	Action Against the Bacteria	Reference
Silver nanoparticles (AgNP)	Bactericidal against *Escherichia coli*, *Porphyromonas gingivalis*, *Aggregatibacter actinomycetememcomitans*	[43]
Copper nanoparticles	Bacteriostatic	[44]
Copper oxide nanoparticles	Bactericidal against methicillin-resistant Staphylococcus aureus (MRSA) and *Escherichia coli*	[51]
Poly lactic-co-glycolic acid (PGLA) with photosensitizer Methylene Blue	Bactericidal against *Enterococcus faecalis*	[60]
Chitosan nanoparticles	Bactericidal against *Enterococcus faecalis*	[61]
Minocycline loaded polyethylene glycol and polylactic acid	Bactericidal against *Aggregatibacter actinomycetememcomitans*	[64]

## Data Availability

Not Applicable.
